# Handheld Dynamometer for Hamstring Strength Test Using Two Different Hand Placements/Methods: An Interrater Reliability Study

**DOI:** 10.1155/2024/9233802

**Published:** 2024-12-10

**Authors:** Thomas A. Koc, Jenna Tucker, Jennifer Gentile, Carla Enriquez, John Lee, Seide Jeanty, Natalia Krasowski

**Affiliations:** Department of Physical Therapy, Kean University, Union, New Jersey 07083, USA

**Keywords:** DPT students, fulcrum, hamstring, proximal

## Abstract

**Background:** Handheld dynamometers provide an accurate measurement of muscle strength and have been shown to have good interrater reliability. The proximal stabilization and fulcrum are two methods of manual muscle testing; however, there is uncertainty about which method may be better for obtaining muscle strength measures.

**Objective:** The purposes were to determine if there was a difference in hamstring strength and to determine the interrater reliability of DPT students using a handheld dynamometer when comparing the proximal stabilization and the fulcrum methods.

**Methods:** A descriptive-comparative research study that examined two methods of manual muscle testing with the use of a Microfet 2 MMT-Wireless digital handheld dynamometer. In prone, each participant was instructed to bend their knee to 90° of knee flexion, where the handheld dynamometer was placed on the lower leg for 5 s. Each technique was performed three times, and an average of the series was calculated.

**Results:** Twenty-nine participants volunteered for this study. The mean scores for Raters 1 and 2 between hamstring testing using the proximal stabilization and fulcrum methods were, respectively, *t*(28) = −2.041, *p* = 0.051, and *t*(28) = −1.990, *p* = 0.056. The interrater reliability showed good reliability between Rater 1 and Rater 2 for hamstring testing for the proximal stabilization method and fulcrum methods, respectively, ICC = 0.742 (95% CI: 0.452, 0.879), *p* ≤ 0.001, and ICC = 0.752 (95% CI: 0.472, 0.884), *p* ≤ 0.001.

**Conclusion:** There are no statistically significant differences between the uses of these two methods in healthy adults; however, there is good interrater reliability of DPT students.

## 1. Introduction

Muscle strength tests are a crucial component of a patient's objective assessment that can divulge information regarding musculoskeletal and neurologic deficits. There are a number of ways to perform muscle strength tests, but the standard method used by physical therapists is the manual muscle test (MMT). During a MMT, the clinician applies manual resistance to the patient's body segment and asks them to push against their force. Generally, the patient is positioned so that the muscle or muscle groups being tested have to move or hold against the resistance. With the information gathered, the clinician will then use clinical judgment to determine a muscle grade for the muscle being tested [1].

Manual muscle testing grades are somewhat subjective in nature. Grading scores range on a 0 to 5 scale, where 0 is *zero strength* and a 5 is considered *normal strength*. Scores are determined by the patient's available range of motion (ROM), whether resistance is held against gravity or in a gravity-minimized position, and how well the patient can hold against a given manual resistance. Though this grading is on a numerical scale, the data collected illustrates ordinal data because the numbers do not represent equal units of measurement [2].

In addition to MMT, muscle strength testing can be conducted using handheld dynamometers (HHDs), which provide a more accurate measurement of the patient's muscle strength. HHDs are portable devices that can be positioned between a clinician's hand and the patient's tested body segment in a similar fashion to MMT performance. The primary difference between the two methods for obtaining measurements is that an MMT is qualitative and a HHD is quantitative. HHDs have been shown to have greater interrater and intrarater reliability when testing positions are standardized among clinicians [3]. Furthermore, a systematic review conducted by Stark et al. [3] concluded that HHDs have similar interrater and intrarater reliability as the gold standard isokinetic dynamometer. Isokinetic dynamometers are considered to be the reference standard for muscle strength measurement due to their ability to obtain vast amounts of information, including peak torque, power, and angle of maximal force. However, HHD is considered to be a more practical means of muscle strength testing in the clinical setting [3].

The objective HHD reading provides excellent reliability and has the potential to detect small changes in muscle strength. Therefore, it can provide valuable information in detecting minimal muscle strength differences [4]. Numerous studies have concluded that HHD is considered to have excellent inter- and intrareliability; however, the inconsistencies in techniques performed leave discrepancies in the literature. A systematic review and meta-analysis conducted by Chamorro et al. [5] reviewed the absolute reliability and concurrent validity of HHD and isokinetic dynamometry. Based on the review, an inconclusive result was collected for both methods due to the lack of consistency in positioning when assessing muscle strength, and when standardized techniques were used, they improved the inter- and intrarater reliability of muscle testing [5].

In textbooks utilized by most physical therapy programs, techniques demonstrate the use of proximal stabilization to obtain measurements [2]. A second reliable technique to collect strength values is referred to as the fulcrum method values [6]. The varying techniques being utilized can create uncertainty and inconsistencies to obtain muscle strength measures within the field of physical therapy.

The two purposes were to determine (1) if there was a significant difference in hamstring muscle strength using a HHD when comparing the proximal stabilization and the fulcrum methods and (2) the interrater reliability of hamstring muscle strength using a HHD by DPT students for both methods.

## 2. Methods

### 2.1. Design

This was a descriptive-comparative research study that examined two different methods of manual muscle testing with the use of a Microfet 2 MMT-Wireless digital HHD. Participants were recruited from a sample of convenience using recruitment flyers from February to June 2022. The study was performed in accordance with the principles of the Declaration of Helsinki and was approved by the Institutional Review Board at Kean University (Federal Registration # IORG 0003969) on December 7, 2021.

### 2.2. Participants and Criteria

The inclusion criteria for this study included individuals who (1) were adult males and females 18 years of age and older without any musculoskeletal conditions within the last 12 months (i.e., fractures and surgeries); (2) were able to read, write, and understand English; and (3) did not experience any pain at rest or pain with movement. The exclusion criteria for this study included individuals who (1) were males and females who were younger than 18 years old; (2) had any integumentary impairments (e.g., open wounds and skin rashes); (3) had any musculoskeletal conditions within the last 12 months (i.e., fractures and surgeries); (4) were not able to read, write, and understand English; and (5) experienced any pain at rest or with movement.

### 2.3. Instrument

Microfet 2 MMT-Wireless digital HHD was used for the assessment of muscle strength. The unit was calibrated by the manufacturer within the allowed limit of ±1% error.

### 2.4. Procedures

**Figure 1:**
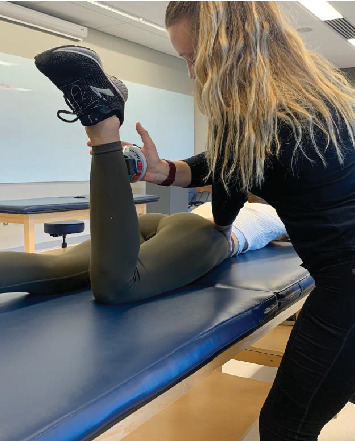
Proximal stabilization method.

**Figure 2:**
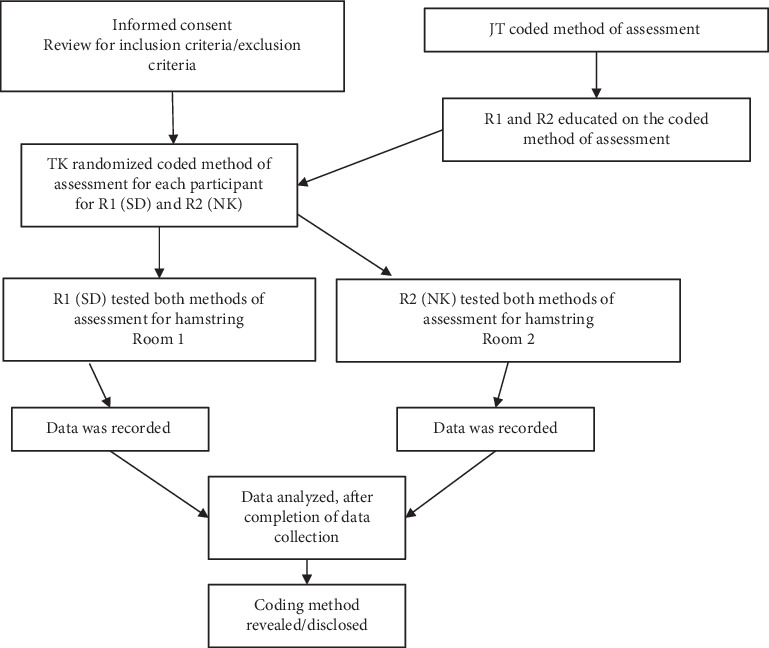
Flow chart of procedures.

Prior to the start of the study, the principal investigator (T.A.K.Jr.) trained both raters (S.J. and N.K.) on the MMT procedures and data collection/recording to maintain consistency throughout testing and minimize potential rater bias. All participants completed an informed consent form that was reviewed for inclusion and exclusion criteria by T.A.K.Jr. One of the secondary researchers (J.T.) coded the method of assessment and informed the two independent raters of the identified code per method of assessment, therefore blinding T.AK.Jr. to the method of assessment. Before each participant's assessment, T.A.K.Jr. randomized the method of assessment and verbalized the coded method to the rater. The rater then completed the assessment for the hamstring muscle groups, visually read the value that was indicated on the HHD, and then recorded the results on a data collection form. The participant then walked around to the other side of an adjoining wall, which separated the lab space between the two raters, where the same procedure was then carried out in the same manner by the second rater. This testing method was performed for each participant. The two raters recorded their findings onto separate data forms, and both raters were blinded to the results of each other's recordings. At the completion of the study following data analysis, the coding method was revealed to T.A.K.Jr. (Figures 1 and 2 and Table 1).

**Figure 3:**
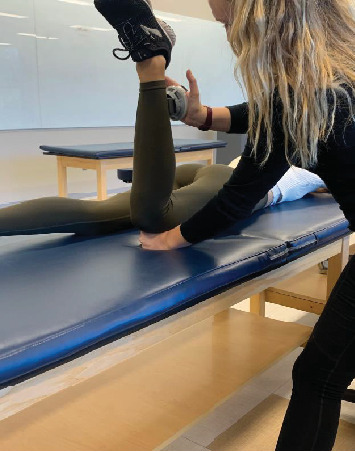
Fulcrum method.

Each participant laid prone on a standard treatment table. In this position, the participant was instructed to bend their knee to 90° of knee flexion, where the Microfet 2 MMT-Wireless HHD was placed on the lower leg, and a downward force toward the table was held for 5 s while the participant resisted the downward force. The participant was instructed to use their maximum effort. Each technique was performed three times, and an average of the series was calculated in pounds of force. For the proximal stabilization method, the proximal stabilization hand was placed at the pelvis (Figure 1), and for the fulcrum method, the hand was placed between the table and the anterior knee (Figure 3).

### 2.5. Data Analysis

Statistical analysis was performed with SPSS Statistics Version 27 (IBM Corporation, Armonk, NY). Descriptive statistics were generated for demographic characteristics. Data were analyzed for normality using the Shapiro–Wilk test. Statistical significance was set at *p* < 0.05 for all analyses. Paired-sample *t*-tests analyzed mean scores for testing the hamstring muscle strength using the standard stabilization method and the fulcrum method. The agreement of the interrater reliability was calculated using intraclass correlation coefficients (ICCs) using a two-way mixed model and consistency type analysis. The values for reliability were interpreted as excellent (0.90), good (0.75–0.89), moderate (0.50–0.74), or poor (< 0.50) [8].

## 3. Results

A total of 29 participants, 13 males (44.8%) and 16 females (55.2%), volunteered to participate in the study. The mean age was 25.9 (SD ± 3.43) and ranged from 22 to 38 years old.

The findings for the Shapiro–Wilk test indicated normally distributed data for Rater 1 for both hamstring testing methods: proximal stabilization method (*W* = 0.976, *p* = 0.728) and fulcrum method (*W* = 0.954, *p* = 0.238) and for Rater 2 for both hamstring testing methods: proximal stabilization method (*W* = 0.970, *p* = 0.570) and fulcrum method (*W* = 0.954, *p* = 0.230).

The mean scores for Rater 1 between hamstring testing proximal stabilization and fulcrum methods (*M* = −1.283, SD ± 3.387) showed no statistical significance between the two methods, *t*(28) = −2.041, *p* = 0.051. The mean scores for Rater 2 between hamstring testing proximal stabilization and fulcrum methods (*M* = −1.6218, SD ± 4.384) showed no statistical significance between the two methods, *t*(28) = −1.990, *p* = 0.056.

The interrater reliability showed good reliability between Rater 1 and Rater 2 for the hamstring testing method and the proximal stabilization method. The average measure ICC_3_ was 0.743 with a 95% confidence interval from 0.452 to 0.879 (*F*(28.28) = 3.884, *p* ≤ 0.001). The interrater reliability showed good reliability between Rater 1 and Rater 2 for the hamstring testing method and the fulcrum method. The average ICC was 0.752 with a 95% confidence interval from 0.472 to 0.884 (*F*(28.28) = 4.032, *p* ≤ 0.001).

## 4. Discussion

In regard to the primary purpose, the results of the study indicated that there are no statistically significant differences between the use of the proximal stabilization versus the fulcrum method when evaluating hamstring muscle strength in healthy individuals. Muscle strength testing is a crucial component of the physical exam that helps guide physical therapists' evaluation and treatment plans. From our results, it may be inferred that one of these MMT methods is not superior compared to the other when assessing muscle strength. A study by Ogborn et al. [9] used other methods of comparing hamstring strength that were performed with the participant in a supine or seated position. The knee was flexed at 90° off of the edge of the table, and a HHD was attached to a cable that was approximated around the participant's ankle [9]. Similarly, in a study by Martín-San Agustín et al. [10], hamstring muscle strength was assessed in a seated position by using a HHD that was attached to a cable that was approximated around the participant's ankle with the knee flexed at 90°; however, this study did not compare different methods of assessment. A study by Kristiansen, Eddy, and Magnusson [11] investigated hamstring isometric strength at the end range using a HHD compared to the Biodex. Based on a thorough literature review, this is the first research study that has investigated the difference between the proximal stabilization versus the fulcrum method when evaluating hamstring muscle strength using a HHD.

In regard to the secondary purpose, the results indicated that there are good reliability agreements between Raters 1 and 2 for both the proximal stabilization and fulcrum methods when evaluating hamstring muscle strength by DPT students. When testing interrater reliability for the knee flexors, Mentiplay et al. [12] reported an ICC of 0.82; Romero-Franco, Jiménez-Reyes, and Montaño-Munuera [13] reported an ICC of 0.994; Kim and Lee [8] reported an ICC of 0.88; Larson, Lorenz, and Melton [14] reported ICC values ranging from 0.84 to 0.96; and a systematic review by Bohannon [15] reported an ICC of 0.72. Albeit there was no mention in these studies about the experience of the raters, our results, focusing on DPT students, also indicate consistent interrater reliability testing with the use of a HDD.

### 4.1. Limitations

There are several limitations to this study. This study only had 29 participants and therefore does not demonstrate generalizability to larger populations. Also, this study only included healthy participants and did not incorporate individuals who sustained or were recovering from a musculoskeletal injury of the lower extremity. There is also a potential for self-selection bias, which is a limitation of this study [16]. The authors also did not control for order effects, in that Rater 1 and Rater 2 remained the same throughout the entire study, and the order of the raters was not randomized.

## 5. Conclusion

To our knowledge, this is the first study to explore the differences between the proximal stabilization and fulcrum methods of MMT using a HHD, which provides evidence that there is no statistically significant difference between the uses of these two methods in healthy adults. Future research studies may be conducted to explore if there are significant differences between the proximal stabilization and fulcrum methods, as well as test the interrater reliability of these methods with those who are recovering from a musculoskeletal injury of the lower extremity. This study also indicates that with adequate training of standardized testing procedures, there is good interrater reliability of DPT students when using a HHD to test hamstring muscle strength.

## Figures and Tables

**Table 1:** Guidelines for reporting reliability and agreement studies (GRRAS) [7].

**Section**	**Page**	**Checklist item**
Title and abstract	1	Identify in the title or abstract that interrater/intrarater reliability or agreement was investigated.
Introduction	4	Name and describe the diagnostic or measurement device of interest explicitly.
		Specify the subject population of interest. Specify the rater population of interest (if applicable).
		Describe what is already known about reliability and agreement and provide a rationale for the study (if applicable).
Methods	6	Explain how the sample size was chosen. State the determined number of raters, subjects/objects, and replicate observations.
		Describe the sampling method.
		Describe the measurement/rating process (e.g., the time interval between repeated measurements, availability of clinical information, and blinding).
		State whether measurements/ratings were conducted independently.
		Describe the statistical analysis.
Results	9	State the actual number of raters and subjects/objects that were included and the number of replicate observations that were conducted.
		Describe the sample characteristics of raters and subjects (e.g., training and experience).
		Report estimates of reliability and agreement including measures of statistical uncertainty.
Discussion/conclusion	10	Discuss the practical relevance of results.

## Data Availability

The data that support the findings of this study are available from the corresponding author upon reasonable request.
